# Effect of therapy switch on time to second-line antiretroviral treatment failure in HIV-infected patients

**DOI:** 10.1371/journal.pone.0180140

**Published:** 2017-07-20

**Authors:** Amanda Häggblom, Michele Santacatterina, Ujjwal Neogi, Magnus Gisslen, Bo Hejdeman, Leo Flamholc, Anders Sönnerborg

**Affiliations:** 1 Unit of Infectious Diseases, Department of Medicine Huddinge, Karolinska Institutet, Karolinska University Hospital, Stockholm, Sweden; 2 Unit of Biostatistics, Institute of Environmental Medicine, Karolinska Institutet, Stockholm, Sweden; 3 Division of Clinical Microbiology, Department of Laboratory Medicine, Karolinska Institutet, Stockholm, Sweden; 4 Department of Infectious Diseases, The Sahlgrenska Academy, University of Gothenburg, Sweden; 5 Department of Infectious Diseases / Venhälsan, South General Hospital, Stockholm, Sweden; 6 Department of Infectious Diseases, Malmö University Hospital, Malmö, Sweden; National and Kapodistrian University of Athens, GREECE

## Abstract

**Background:**

Switch from first line antiretroviral therapy (ART) to second-line ART is common in clinical practice. However, there is limited knowledge of to which extent different reason for therapy switch are associated with differences in long-term consequences and sustainability of the second line ART.

**Material and methods:**

Data from 869 patients with 14601 clinical visits between 1999–2014 were derived from the national cohort database. Reason for therapy switch and viral load (VL) levels at first-line ART failure were compared with regard to outcome of second line ART. Using the Laplace regression model we analyzed the median, 10^th^, 20^th^, 30^th^ and 40^th^ percentile of time to viral failure (VF).

**Results:**

Most patients (n = 495; 57.0%) switched from first-line to second-line ART without VF. Patients switching due to detectable VL with (n = 124; 14.2%) or without drug resistance mutations (DRM) (n = 250; 28.8%) experienced VF to their second line regimen sooner (median time, years: 3.43 (95% CI 2.90–3.96) and 3.20 (95% 2.65–3.75), respectively) compared with those who switched without VF (4.53 years). Furthermore level of VL at first-line ART failure had a significant impact on failure of second-line ART starting after 2.5 years of second-line ART.

**Conclusions:**

In the context of life-long therapy, a median time on second line ART of 4.53 years for these patients is short. To prolong time on second-line ART, further studies are needed on the reasons for therapy changes. Additionally patients with a high VL at first-line VF should be more frequently monitored the period after the therapy switch.

## Introduction

Antiretroviral therapy (ART) has substantially reduced mortality and morbidity in individuals with human immunodeficiency virus type 1 (HIV-1) infection [1]. However, patients frequently switch to alternate drug combinations due to toxicity, convenience or costs [2–4], but also due to virological treatment failure. Reappearance of HIV RNA in plasma may or may not be associated with drug resistance mutations (DRM). Lack of DRM is frequently due to poor adherence, but is also due to a high genetic barrier to resistance for some drugs [5, 6]. In addition, standard genotypic resistance testing (GRT) may underestimate drug resistance[7] and DRM in minor quasispecies can contribute to treatment failure[8].

To date, cohort studies have reported partly conflicting results on the consequences for disease progression by the various patterns of drug resistance at treatment failure [9–13]. The reasons for these inconsistencies remain unclear [12], but the limited time of follow-up in most studies should be noted. Also, it is well known that the viral load (VL) level at treatment initiation plays a determinative role in the first-line treatment response and the development of DRM [14].

The aim of the study was to analyze the consequences of different reason for therapy switch from first line ART on second-line ART outcome, using the Swedish cohort which represents a highly diversified HIV epidemic in a real-life setting [15]. This was done by analyzing the time to second-line viral failure (VF) and the increase of CD4+ T-cells at 12 and 24 months of second-line ART. Moreover, since the baseline level of VL at initiation of first-line ART is an independent factor associated with decreased virological success [14], the effect of VL level at first-line ART failure on the second line outcome was investigated. For all analysis, patients were included over a period of 15 years, 1999–2014.

## Material and methods

### Study population

**Fig 1:**
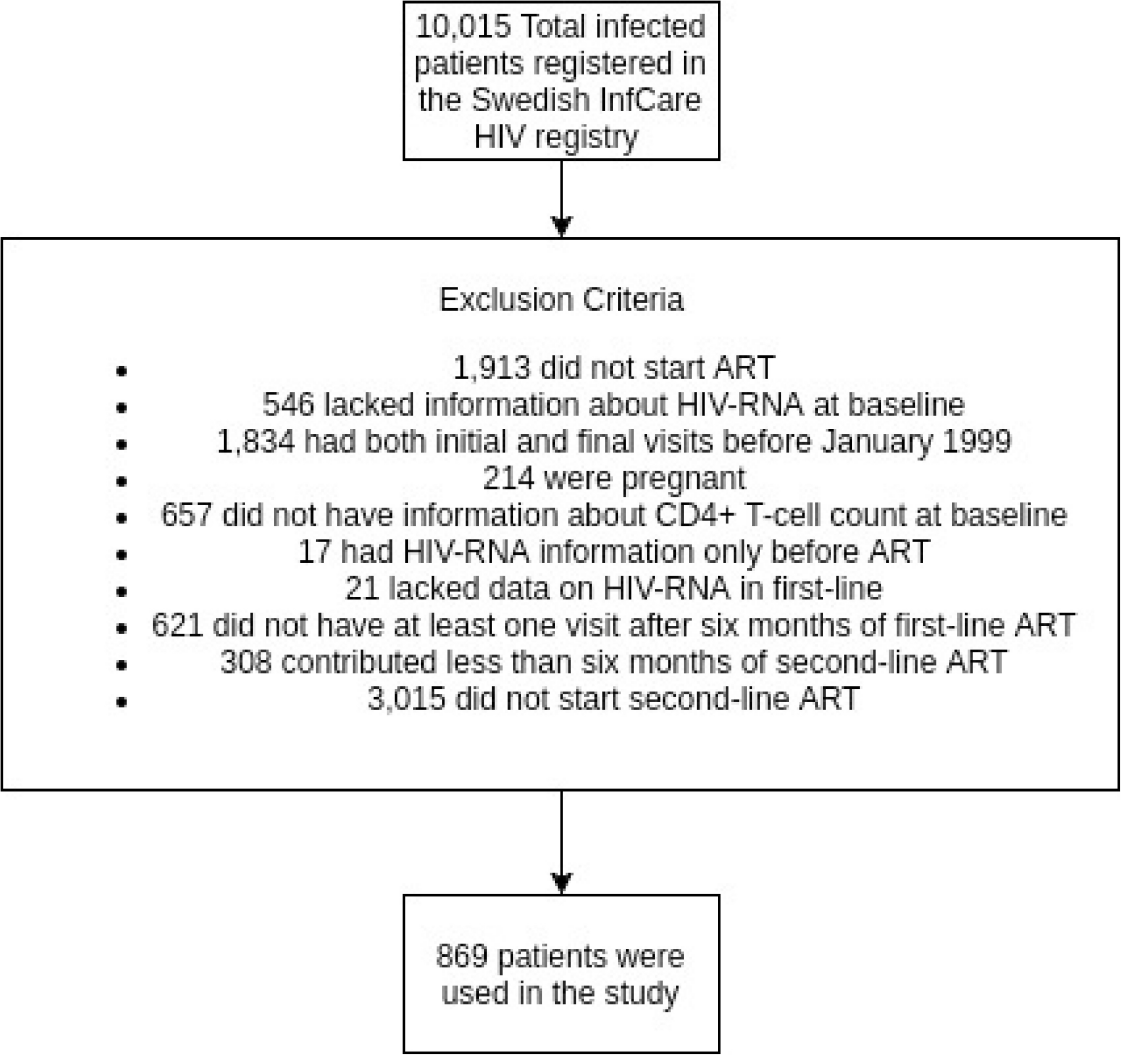
Flow chart of patients exclusions from the study.

Our study used observational data from the Swedish InfCare HIV database, which collects data through a clinical decision support tool and includes >99% of living HIV infected patients and the majority of patients diagnosed between 1983–2014 at 30 infectious disease clinics from all regions of the country [15]. As of January 2015, a total of 10,015 HIV-infected patients were registered. Of these, 8,102 (81%) patients had started ART. Patients who were alive, not pregnant at ART initiation, under follow up after January 1999, and with a switch to second-line ART, were eligible. Baseline visit was set as first available visit after the therapy switch. During follow-up, patients contributed with a minimum of two and a maximum of 52 visits. Each patient enrolled, contributed from baseline visit until: (1) end of second-line ART (if started); (2) date of death or (3) January 2015 (end of follow-up). Data on socio-demographic characteristics, VL (if detectable) and CD4^+^ T-cell counts, HIV-1 subtypes, type of ART, and presence and type of any HIV DRM (if detectable) were collected. ART was classified as: non-nucleoside reverse transcriptase inhibitor (NNRTI) based, ritonavir-boosted protease inhibitor (PI/r) based, protease inhibitor without boosting (PI), and other. A total of 869 patients and 14601 clinical visits were included in the study see Fig 1 for exclusion criterias. The Regional Ethical Review Boards in Stockholm and Gothenburg have approved the research (2005/1167–31/3; 2011-06-20, Dnr: 532–11).

### Outcome variables

Primary outcome was time to viral failure (VF) after second-line ART initiation. Therapy switch from first to second-line ART was defined as any change between (1) NNRTI and PI or (2) change of any NRTI, PI or NNRTI while remaining on PI or NNRTI based ART regimen. Viral failure was defined as one VL> = 200 copies/mL after at least six months of a new ART line initiation (first-line or second-line ART). Sensitivity analysis were done using different definitions of VF: 1) one VL> = 50 copies/mL; 2) two consecutive VL> = 50 copies/mL after six months and nine months, respectively, of second-line ART initiation, with no differences in the estimates (data not shown). We controlled the frequency of VL measurements in our study population and all patients had at least one VL measurement per year. On average, each patient had 5 VL measurements per year.

Secondary outcomes were: (1) median CD4+ T-cell counts at 12 and 24 months of second-line ART; (2) time to death and (3) time to AIDS.

### Patient categories at switch from first to second-line ART

Switch to second-line ART was categorized into three different categories: (1) switch without VF and therefore GRT was not performed; (2) switch due to VF and no detectable DRM at standard GRT; (3) switch due to VF and detectable DRM.

Real time PCR RNA quantification tests (COBAS® AmpliPrep/COBAS® TaqMan® HIV-1 Test) were used for VL monitoring. VL at second-line ART initiation was categorized in six categories: (1) 0–200; (2) 200–500; (3) 501–1000; (4) 1,001–10,000; (5) 10000–100 000 and (6) 100001+ HIV RNA copies/mL.

### Other potential confounders

Potential confounders for time to second-line VF and changes in CD4+ T-cell counts were chosen as: VL (< = 100.000; >100.000 copies/mL), and CD4+ T-cell count (<200; 200–350; 350–500; and >500 cells/mL) at baseline and at second-line ART initiation; age (0–30; 31–40; 41–50; >50 years) at first-line ART initiation; type of ART regimen (NNRTI based, PI/r based, PI based, and Other) at first and second-line ART; country of birth (Sweden vs Non-Sweden); gender (Female; Male); route of transmission (heterosexual, men having sex with men (MSM), people who inject drugs (PWID), other); and follow-up time on first-line ART as a continuous variable, and time on second-line ART as a continuous variable.

### Genotype resistance

Any DRM detected appearing at first line ART failure and before switch were described. In case of several tests, the closest one before switch was used. Thus, this test showed all DRM which had appeared from start of virological treatment failure. Of the total number of patients included, 479 (56%) had a DRM test at viral failure.

Viral nucleotide sequence data was submitted to the Stanford University HIV Drug Resistance Database (HIVdb) [16] using Sierra. DRM were defined according to the IAS-USA 2014 list [17]. The level of clinically relevant resistance for each drug was determined using the Stanford HIVdb algorithm [18].

### Statistical analysis

Laplace regression is a statistical method which estimate multivariable survival percentiles and evaluate the effects of exposures on them. Survival percentiles are important summary measures of a time-to-event outcome of interest. For instance, in this study, the 50th survival percentile is that value of time to VF of second-line ART for which 50% of the study individuals have a value below it and 50% above it. Using Laplace regression we were able to estimate the effect of switch to second-line ART on different percentiles of time to VF of second-line ART adjusting for potential confounders. More specifically, assume that a significative effect of switch to second-line ART on median time to VF of second-line ART is found. This means that the median time to VF of second-line ART across categories of switching to second-line ART varies significatively, i.e. switching has an effect on median time to VF. Similar interpretation can be done with all other survival percentiles. The median, 10^th^, 20^th^, 30^th^ and 40^th^ percentiles of time to VF of second-line ART, death and AIDS were modeled by using a Laplace regression model [19] adjusting for the described relevant factors. Median CD4^+^ T-cell count at 12 and 24 months second-line ART was modeled by using a quantile regression model [20] adjusted for the relevant factors. Not all patients had a CD4^+^ T-cell count in the interval of 11 to 13 months or in the interval of 23 to 25 months of second-line ART, so there was a high rate of missing values. To take that into account, we conducted a weighted analysis [21] on median CD4^+^ T-cell count at 12 (11–13 month) and 24 months (23–25 month) with no changes on the estimates (data not shown). Weights were estimated modeling the probability of having a missing CD4^+^ T-cell count at 12 (11–13) or 24 (23–25) months separately and taking its inverse.

## Results

### Patient characteristics

Baseline demographic and laboratory characteristics of the study population (n = 869) are described in Table 1. Tables 1 and 2 contains a detailed description of the population by categories of therapeutic switch and VL level at second-line ART initiation.

**Table 1:** Demographic and clinical characteristics of the patients by category of antiretroviral therapy (ART) switch.

	No failure; n (%)	Viral failure only; n (%)	Viral failure with DRM; n (%)	Total; n (%)
**Number of patients**	495 (57)	250 (28.8)	124 (14.2)	869 (100)
**Sex**				
Female	176 (50.4)	116 (33.2)	57 (16.3)	349 (100)
Male	319 (61.3)	134 (25.8)	67 (12.9)	520 (100)
Total	495 (57)	250 (28.8)	124 (14.3)	869 (100)
**First-line ART**				
NNRTI based	99 (47.8)	64 (30.9)	44 (21.3)	207 (100)
New drugs*	1 (100)	0 (0)	0 (0)	1 (100)
Other	57 (64)	28 (31.5)	4 (4.5)	89 (100)
PI unboosted	73 (41)	70 (39.3)	35 (19.7)	178 (100)
PI/r based	265 (67.3)	88 (22.3)	41 (10.4)	394 (100)
Total	495 (57)	250 (28.8)	124 (14.3)	869 (100)
**Second-line ART**				
NNRTI based	223 (61.3)	103 (28.3)	38 (10.4)	364 (100)
Other	116 (68.2)	28 (16.5)	26 (15.3)	170 (100)
PI unboosted	30 (69.8)	12 (27.9)	1 (2.3)	43 (100)
PI/r based	126 (43.2)	107 (36.6)	59 (20.2)	292 (100)
Total	495 (57)	250 (28.8)	124 (14.3)	869 (100)
**Route of transmission**				
PWID	17 (34)	26 (52)	7 (14)	50 (100)
Heterosexual	249 (54.5)	135 (29.5)	73 (16)	457 (100)
MSM	185 (69.8)	61 (23)	19 (7.2)	265 (100)
Other	40 (44.0)	27 (29.7)	24 (26.4)	91 (100)
Total	491 (56.9)	249 (28.9)	123 (14.3)	863 (100)
**Age in years**				
0–30	100 (47.8)	64 (30.6)	45 (21.5)	209 (100)
31–40	184 (56.1)	104 (31.7)	40 (12.2)	328 (100)
41–50	129 (63.5)	49 (24.1)	25 (12.3)	203 (100)
>50	82 (63.6)	33 (25.6)	14 (10.9)	129 (100)
Total	495 (57.0)	250 (28.8)	124 (14.3)	869 (100)
**Country of birth**				
Sweden	222 (62.7)	100 (28.2)	32 (9)	354 (100)
Africa	156 (50.2)	91 (29.3)	64 (20.6)	311 (100)
Asia	66 (55.9)	36 (30.5)	16 (13.6)	118 (100)
Latin America	21 (60)	10 (28.6)	4 (11.4)	35 (100)
Others	28 (62.2)	12 (26.7)	5 (11.1)	45 (100)
Total	493 (57.1)	249 (28.9)	121 (14)	863 (100)
**CD4 cell count at baseline (first visit from 1999) (cells/ul)**				
<200	142 (60.9)	52 (22.3)	39 (16.7)	233 (100)
200–349	187 (60.9)	83 (27.0)	37 (12.1)	307 (100)
350–499	102 (52.8)	63 (32.6)	28 (14.5)	193 (100)
500+	64 (47.1)	52 (38.2)	20 (14.7)	136 (100)
Total	495 (57.0)	250 (28.8)	124 (14.3)	869 (100)
**CD4 cell count at second-line ART initiation (cells/ul)**				
<200	27 (35.1)	32 (41.6)	18 (23.4)	77 (100)
200–349	104 (46.8)	78 (35.1)	40 (18.0)	222 (100)
350–499	133 (59.1)	64 (28.4)	28 (12.4)	225 (100)
500+	231 (67.0)	76 (22.0)	38 (11.0)	345 (100)
Total	495 (57.0)	250 (28.8)	124 (14.3)	869 (100)
**HIV-1 RNA at baseline (first visit from 1999) (copies/ml)**				
< = 100.000	461 (58.4)	220 (27.9)	108 (13.7)	789 (100)
100.000+	34 (42.5)	30 (37.5)	16 (20.0)	80 (100)
Total	495 (57.0)	250 (28.8)	124 (14.3)	869 (100)
**HIV-1 RNA at second-line ART initiation (copies/ml)**				
< = 100.000	494 (57.9)	238 (27.9)	121 (14.2)	853 (100)
100.000+	1 (6.3)	12 (75.0)	3 (18.8)	16 (100)
Total	495 (57.0)	250 (28.8)	124 (14.3)	869 (100)
**Years on first-line ART**	2.74 (2.25)	3.46 (2.54)	3.6 (2.74)	3.1 (2.4)
**Years on second-line ART**	2.65 (1.94)	2.6 (2.12)	2.01 (1.94)	2.5 (2)
**Years of follow up**	5.79 (2.88)	6.44 (3.35)	5.99 (3.38)	6.0 (3.1)

*New drugs refers to the drugs all integrase inhibitors, rilpivirine or maraviroc

**Table 2:** Demographic and clinical characteristics by category of viral load (VL) at second-line antiretroviral therapy (ART) initiation.

Viral load	0–200	200–500	501–1000	1000–10000	10k-100k	100k+	Total
	728(83.77)	46(5.29)	21 (2.42)	42 (4.83)	16 (1.84)	16 (1.84)	869 (100)
**Sex**														
Female	284 (81.4)	11 (3.2)	12 (3.4)	21 (6)	12 (3.4)	9 (2.6)	349 (100)
Male	444 (85.4)	35 (6.7)	9 (1.7)	21 (4)	4 (0.8)	7 (1.3)	520 (100)
Total	728 (83.8)	46 (5.3)	21 (2.4)	42 (4.8)	16 (1.8)	16 (1.8)	869 (100)
**First-line ART**														
NNRTI	158 (76.3)	17 (8.2)	9 (4.3)	11 (5.3)	5 (2.4)	7 (3.4)	207 (100)
New drugs*	1 (100)	0 (0)	0 (0)	0 (0)	0 (0)	0 (0)	1 (100)
Other	78 (87.6)	4 (4.5)	1 (1.1)	2 (2.2)	3 (3.4)	1 (1.1)	89 (100)
PI	139 (78.1)	12 (6.7)	5 (2.8)	14 (7.9)	4 (2.2)	4 (2.2)	178 (100)
PI/r	352 (89.3)	13 (3.3)	6 (1.5)	15 (3.8)	4 (1)	4 (1)	394 (100)
Total	728 (83.8)	46 (5.3)	21 (2.4)	42 (4.8)	16 (1.8)	16 (1.8)	869 (100)
**Second-line ART**														
NNRTI based	322 (88.5)	17 (4.7)	6 (1.6)	12 (3.3)	5 (1.4)	2 (0.5)	364 (100)
Other	154 (90.6)	5 (2.9)	1 (0.6)	5 (2.9)	2 (1.2)	3 (1.8)	170 (100)
PI	32 (74.4)	4 (9.3)	0 (0)	4 (9.3)	2 (4.7)	1 (2.3)	43 (100)
PI/r	220 (75.3)	20 (6.8)	14 (4.8)	21 (7.2)	7 (2.4)	10 (3.4)	292 (100)
Total	728 (83.8)	46 (5.3)	21 (2.4)	42 (4.8)	16 (1.8)	16 (1.8)	869 (100)
**Route of transmission**														
PWID	35 (70)	5 (10)	1 (2)	6 (12)	3 (6)	0 (0)	50 (100)
Hetero.	378 (82.7)	24 (5.3)	13 (2.8)	26 (5.7)	7 (1.5)	9 (2)	457 (100)
MSM	237 (89.4)	13 (4.9)	5 (1.9)	5 (1.9)	1 (0.4)	4 (1.5)	265 (100)
Other	74 (81.3)	3 (3.3)	2 (2.2)	4 (4.4)	5 (5.5)	3 (3.3)	91 (100)
Total	724 (83.9)	45 (5.2)	21 (2.4)	41 (4.8)	16 (1.9)	16 (1.9)	863 (100)
**Age in years**														
0–30	169 (80.9)	8 (3.8)	9 (4.3)	12 (5.7)	4 (1.9)	7 (3.3)	209 (100)
31–40	278 (84.8)	15 (4.6)	7 (2.1)	17 (5.2)	6 (1.8)	5 (1.5)	328 (100)
41–50	174 (85.7)	15 (7.4)	3 (1.5)	9 (4.4)	1 (0.5)	1 (0.5)	203 (100)
>50	107 (82.9)	8 (6.2)	2 (1.6)	4 (3.1)	5 (3.9)	3 (2.3)	129 (100)
Total	728 (83.8)	46 (5.3)	21 (2.4)	42 (4.8)	16 (1.8)	16 (1.8)	869 (100)
**Country of birth**									
Swed.	305 (86.2)	25 (7.1)	6 (1.7)	9 (2.5)	7 (2)	2 (0.6)	354 (100)
Africa	252 (81)	14 (4.5)	10 (3.2)	21 (6.8)	7 (2.3)	7 (2.3)	311 (100)
Asia	97 (82.2)	3 (2.5)	3 (2.5)	10 (8.5)	1 (0.8)	4 (3.4)	118 (100)
Latin	32 (91.4)	0 (0)	0 (0)	1 (2.9)	0 (0)	2 (5.7)	35 (100)
Others	37 (82.2)	4 (8.9)	2 (4.4)	1 (2.2)	0 (0)	1 (2.2)	45 (100)
Total	723 (83.8)	46 (5.3)	21 (2.4)	42 (4.9)	15 (1.7)	16 (1.9)	863 (100)
**Categories of CD4 T- cell count at baseline (first visit from 1999)**														
<200	199 (85.4)	11 (4.7)	2 (0.9)	14 (6)	4 (1.7)	3 (1.3)	233 (100)
200–349	257 (83.7)	17 (5.5)	9 (2.9)	12 (3.9)	4 (1.3)	8 (2.6)	307 (100)
350–499	159 (82.4)	10 (5.2)	4 (2.1)	11 (5.7)	7 (3.6)	2 (1)	193 (100)
500+	113 (83.1)	8 (5.9)	6 (4.4)	5 (3.7)	1 (0.7)	3 (2.2)	136 (100)
Total	728 (83.8)	46 (5.3)	21 (2.4)	42 (4.8)	16 (1.8)	16 (1.8)	869 (100)
**Categories of CD4 T-cell count at second-line ART initiation**														
<200	50 (64.9)	6 (7.8)	2 (2.6)	7 (9.1)	5 (6.5)	7 (9.1)	77 (100)
200–349	157 (70.7)	13 (5.9)	7 (3.2)	28 (12.6)	10 (4.5)	7 (3.2)	222 (100)
350–499	192 (85.3)	20 (8.9)	6 (2.7)	6 (2.7)	0 (0)	1 (0.4)	225 (100)
500+	329 (95.4)	7 (2)	6 (1.7)	1 (0.3)	1 (0.3)	1 (0.3)	345 (100)
	728 (83.8)	46 (5.3)	21 (2.4)	42 (4.8)	16 (1.8)	16 (1.8)	869 (100)

*New drugs refers to the drugs all integrase inhibitors, rilpivirine or maraviroc

### Reason for therapy switch from first-line therapy and DRM at switch

Out of the 869 patients, 495 (57.0%) switched to second-line ART without a virologic failure, 250 (28.8%) switched with a VF without DRM, and 124 (14.2%) switched with a virological failure and PI, NNRTI and/or NRTI DRM.

A total of 207 patients on NNRTI based first-line ART switched to second-line ART of whom 99 (47.8%) switched without a VF, 64 (30.9%) switched with VF without any DRM, and 44 (21.3%) with VF and DRM. For the 394 patients on a boosted PI based first-line ART, the number of individuals in the same categories of switches were: 265 (67.3%), 88 (22.3%), and 41 (10.4%), respectively. For the 178 patients on an unboosted PI based first-line ART, the number of individuals in the same categories of switches were: 73 (41.0%), 70 (30.9%), and 35 (19.7%), respectively. Eighty-six patients started other ART regimens (Table 1).

### DRM at second-line treatment failure

An NNRTI-based regimen was given as second-line in 364 patients, 292 were given PI/r based regimen, 43 an unboosted PI based regimen and 170 other treatments. At treatment failure, 287 DRM occurred among 85 patients and the most common were: M184I/V (n = 65, % = 22.6), K103N (n = 51, % = 17.8), V108I (n = 14, % = 4.9), D67N (n = 12, % = 4.2), K70R (n = 12, % = 4.2), M41L (n = 11,% = 3.8), K101E (n = 7, % = 2.4), T215F (n = 7, % 2.4), Y181C (n = 7,% = 2.4).

### Effect of reason to switch on time to failure of the second-line ART

**Fig 2:**
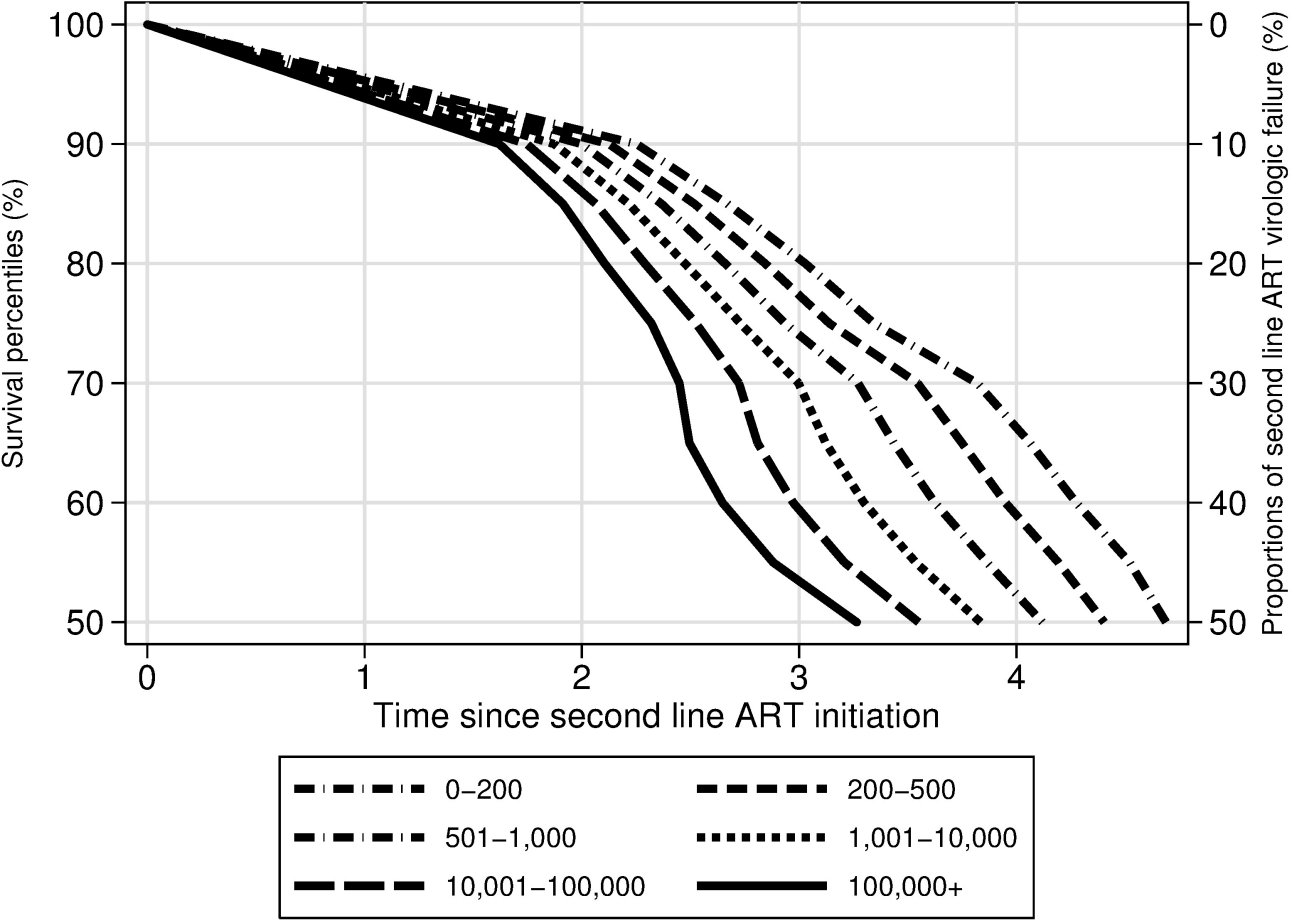
Predicted survival percentiles (1–50%) of second-line ART viral failure stratified by reason to therapy switch. Predicted survival percentiles of time to VF of second-line ART modeled using a Laplace regression adjusted by: Sex, type of regimen first and second-line ART, route of transmission, country of birth. Age at first-line ART initiation, CD4 cell count at first and second-line ART initiation, HIV RNA load at first and second-line ART initiation, time in first-line ART and time in second-line ART as continuous variables. DRM = drug resistance mutations.

Patients switching from first-line to second-line ART due to virological failure with (n = 124) or without (n = 250) any DRM experienced VF to their second line regimen sooner compared to second-line VF in all studied survival percentiles compared with patients switching without failure (n = 495). For example, the first 50% (median) of virologic failures occurred within 4.53 years of second-line ART among patients who switched first line ART without failure (Reference group) and within 3.43 years (1.1 year before) among patients who switched due to virological failure and at least one DRM. Fig 2 depicts predicted survival percentiles (1–50%) of second-line ART failure stratified by type of switch. There was no significant difference in time to second-line treatment failure between failure due to a detectable VL only and failure with DRM (data not shown). The number of patients failing second-line therapy among patients without detectable VL at switch was 45 (9%). The number patients with a detectable VL but without any DRM was 83 (33%). The number of patinents with at least one DRM was 34 (27%). Detailed information of effect of reason to switch on time to failure of the second-line ART is given in the supplementary materials (S1 Table).

### Effect of VL level at second-line ART initiation on time to second-line ART failure

**Fig 3:**
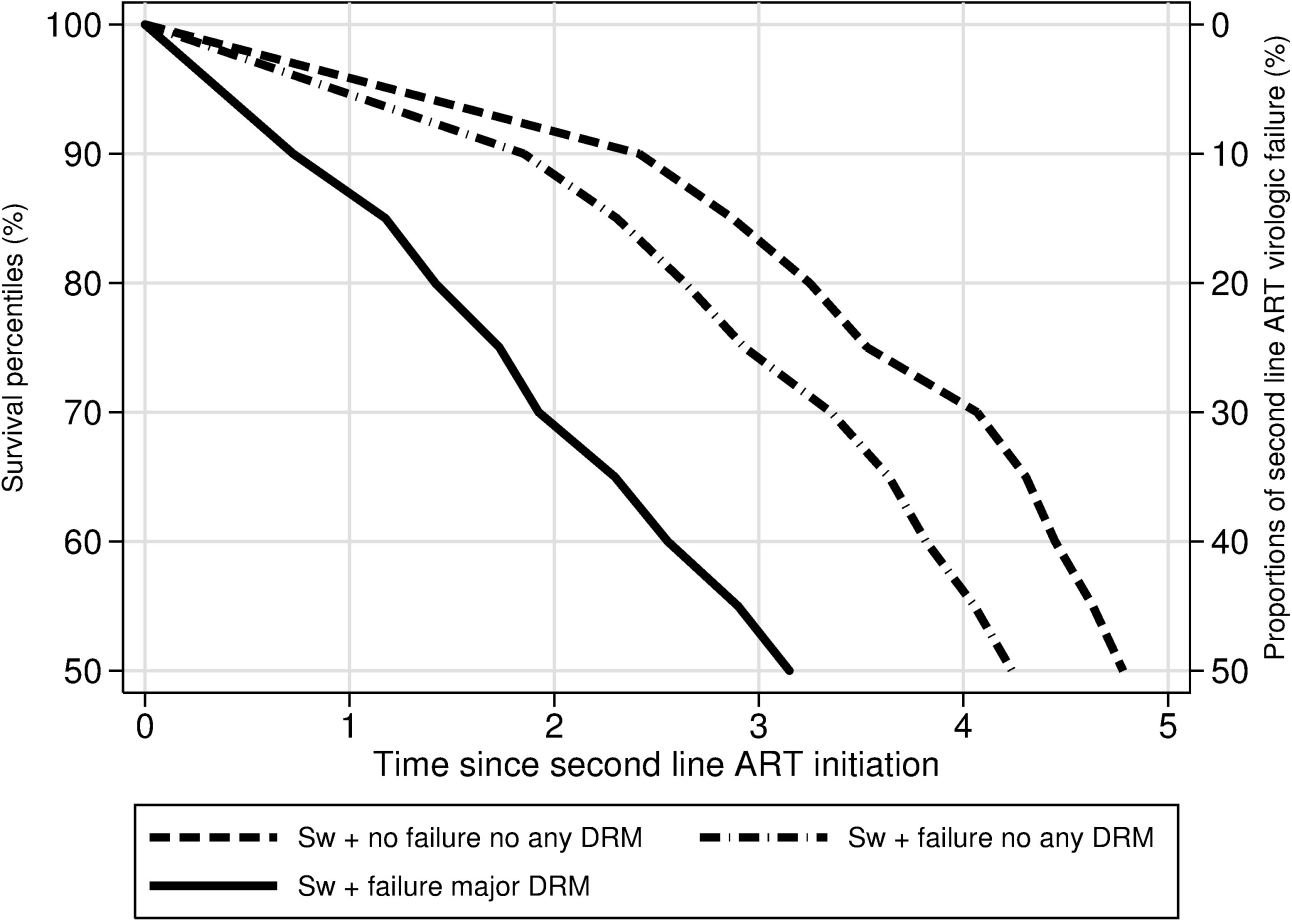
Predicted survival percentiles (1–50%) of second-line ART virological failure by category of HIV-1 RNA load at second-line ART initiation. Predicted survival percentiles of time to VF of second-line ART modeled using a Laplace regression adjusted by: Sex, type of regimen first and second-line ART, route of transmission, country of birth. Age at first-line ART initiation, CD4 cell count at first and second-line ART initiation, HIV RNA load at first and second-line ART initiation, time in first-line ART and time in second-line ART as continuous variables.

Patients initiating second-line ART with VL 201–500, 501–1.000, 1.001–10.000, 10.001–100.000 and >100.000 copies/mL, respectively, experienced VF to their second line regimen sooner in the 30^th^, 40^th^ and median survival percentile compared with patients who initiated it with a VL between 0 and 200 copies/mL. Nonetheless, no clear difference was seen among the failures that occurred in the lower percentile (10^th^ and 20^th^). For example, the first 50% (median) of second-line ART failures occurred within 4.68 years for the patients with a VL 0–200 copies/ml at first-line treatment failure (reference group). For patients switching with a VL of 201–500 copies/ml it occurred within 3.86 years (-0.82 years before (95% CI-1.51; -0.13 years)). Fig 3 reports the predicted values of survival percentiles (10–50%) by category of VL at second-line ART initiation. Detailed information of effect of VL level at second-line ART initiation on time to second-line ART failure is given in the supplementary materials (S2 Table).

### Secondary outcomes: CD4+ T-cell counts at 12 and 24 months, time to AIDS and death

Type of switch and VL at second-line ART initiation did not show any significant effect on median CD4+ T-cell counts at 12 and 24 months, respectively (S3 and S4 Tables). Out of a total of five AIDS events only after second-line ART initiation, all of them occurred in patients with failure and no DRM. Out of a total 38 deaths after second-line ART initiation, 12 (32%) occurred among patients with no failure, 22 (58%) with failure but no DRM, and four (10%) with failure plus DRM. Fourteen of the 38 deaths were AIDS related. No effect of switch and VL at second-line ART initiation was found on time to AIDS or death (data not shown). Nonetheless, given the low sample size, inferences might be not precise.

## Discussion

In this study we analyzed the effect of different causes to first line ART switch, and the level of VL at switch, on the long-term outcome of second-line treatment using the InfCare cohort, which represents >99% of all diagnosed HIV infected patients in Sweden. The majority of the patients switched therapy due to other reasons than VF with no difference over the calendar years (data not shown). This consistent pattern of reason for therapy switch could be due to that although the virological treatment failure was more common in the past, switches due to side effects of the older drugs also occurred to a higher extent. Our data thus confirm that toxicity and/or convenience are by far the most common reason for therapy switch of first-line ART in a real-life situation in a high-income country.

Among the patients who had a detectable VL at treatment failure, the vast majority did not have any DRM and in total only 65 (8%) of the patients switching from first to second-line ART had one or more DRM. The low rate of DRM among patients with detectable VL could be due to either a poor adherence or use of drugs with a high genetic barrier, the latter illustrated by the low proportion (10.4%) of DRM in patients with PI/r based ART as compared to NNRTI-based ART (21.3%) and unboosted PI (19.7%). However, once VF occurred, it had a significantly negative effect on time to second-line VF regardless of DRM status. The significant differences started to appear around 2.5 years. The median time to failure was 4.3 years of second-line treatment for patients with no detectable VL at switch compared to 3.2 (95% 2.65–3.75) years for patients with VF only and 3.43 (95% CI 2.9–3.96) years for patients with at least one DRM at failure. Several factors can explain this finding. For example at virological failure the reservoirs are reseeded rapidly resulting in a high viral burden to contain with the new treatment. Also, there may be drug resistant quasispecies present that are not detected by routine resistance testing[22]. In addition, the reason to a first-line treatment failure is frequently suboptimal adherence and such behavioral characteristics of a failing patient may persist despite increased adherence support[23].The choice of the 2nd line regimen was made by the clinicians in a real-world setting where the GRT results are one of several parameters to consider at the choice. Therefore, we did not include predicted activities of the individual drugs in the study. However the impact of the predicted activity following a detected DRM would be of interest to include in future research. It is well known that the VL at baseline in patients who are given first-line therapy is an independent factor of treatment failure and of time to virologic suppression[24, 25]. In clinical trials, the patients are frequently stratified based on a VL above or below 100000 copies/ml when evaluating the results[26–28]. Also, patients with a VL > 500.000/copies/ml have been reported to deserve special attention [14]. Patients in our cohort generally started second-line with a VL < 100.000/copies/ml. Despite this fact higher VL was significantly associated with shorter time to virological treatment failure. Detectable VL (>400 copies/ml) at first-line treatment failure has been shown to be predictive for poorer outcome after switch [25, 26]. However, to our knowledge our study is the first with a detailed analysis of differences in time to VF for several levels of VL at switch to second-line treatment, suggesting a clinical value to detect viral rebound at an early stage. In contrast, the reasons of switch and the VL at second-line ART initiation did not show any significant effect on median CD4^+^ T-cell counts at 12 and 24 months of second-line ART and also no effect on the few AIDS and death cases.

Partly in contrast to our results a study from Italy with GRT performed between 1998–2004, showed that three classes DRM was associated with clinical progression only when the model was not adjusted for calendar years, whereas another Italian study performed between 1999–2003 could establish the relationship (also when adjusting for calendar year). The FIRST study showed that NNRTI-DRM is the strongest predictor of poor clinical outcome [27]. Furthermore in conflict to our results several studies have documented a poor clinical outcome in general among patients with DRM [13, 28] and that the association could not be explained by differences in CD4+ T-cell count or HIV RNA levels [29]. The most recent study showed slightly less steep CD4+ T-cell declines among patients with DRM however also stated it might be due to unmeasured factors such as poor adherence[30]. A reason for these inconsistencies in the results remains still unclear, although it might depend on the study design, the statistical approach or on the characteristics of the cohorts [12]. Also studies performed within the recent five years are lacking. Most likely the reason for poor prognosis among patients with DRM is that those studies were performed during the in early years of ART were fewer treatment options were available.

In summary since our study includes patients from 1999 until 2014 and adjusts for time in follow up, it reflects on the current clinical practice and state of the art antiretroviral regimens, although no patients with integrase inhibitors were included. Treatment modifications were commonly done due to other reasons than viral rebound. The different reasons for therapy switch studied are not related to poor CD4+ T-cell gain on second-line treatment. In the context of life-long therapy, the median time on second line ART of 4.53 years is short. To improve time on second-line therapy further evaluations are needed of the reasons to therapy switch if patients have an undetectable VL. Furthermore the patients with a high viral load at first-line treatment failure should be more frequently monitored the time period after therapy switch.

## Supporting information

S1 Table Effect of categories of therapy switch to second-line ART on time in years to second-line ART virological failure at 10th, 20th, 30th, 40th and median survival time. (DOCX)

S2 Table Effect of categories of HIV RNA load at second-line ART initiation on time in years to second-line ART virological failure at 10^th^, 20^th^, 30^th^, 40^th^ and median survival time. (DOCX)

S3 Table Estimates of the effect of therapeutic switch on CD4 cell count at 12 and 24 months since second line ART initiation. (DOCX)

S4 Table Estimates of the effect of viral load at second line ART initiation on CD4 cell count at 12 and 24 months. (DOCX)
