# Supplementary material for: Effect of therapy switch on time to second-line antiretroviral treatment failure in HIV-infected patients
Source: PLoS One. 2017 Jul 20;12(7):e0180140. doi: 10.1371/journal.pone.0180140 (PMC5519043; doi:10.1371/journal.pone.0180140)
Supplement: S1 Table — (DOCX) [file pone.0180140.s001.docx]

**Table S1. Effect of categories of therapy switch to second-line ART on time in years to second-line ART virological failure at 10th, 20th, 30th, 40th and median survival time (Sweden 1999-2015, n=869).**

| **Therapeutic switch** | **10th survival percentile** | **20th survival percentile** | **30th survival percentile** | **40th survival percentile** | **Median survival percentile** |
| --- | --- | --- | --- | --- | --- |
| No failure | 2.35^a^ | 3 | 3.65 | 4.1 | 4.53 |
| Failure no DRM^c^ | -1.26  (-2.35; -0.16)^b^ | -1.55  (-2.12; -0.99) | -1.51  (-2.08; -0.94) | -1.46  (-2.18; -0.74) | -1.33  (-1.88; -0.78) |
| Failure + DRM | -1.07  (-2.2; 0.06) | -1.33  (-2.03; -0.62) | -1.27  (-2.06; -0.49) | -1.19  (-1.84; -0.53) | -1.1  (-1.63; -0.57) |

Intercept coefficient with time in second-line ART and time in follow up centered in their mean. All the remaining covariates equal to reference group. Adjusted by: sex, type of regimen first and second-line ART, route of transmission, country of birth. Age at first-line ART initiation, CD4 cell count at first and second-line ART initiation, HIV RNA load at first and second-line ART initiation, time in first-line ART, time in second-line ART and time in follow up.DRM= drug resistance mutations.
