# Supplementary material for: Effect of therapy switch on time to second-line antiretroviral treatment failure in HIV-infected patients
Source: PLoS One. 2017 Jul 20;12(7):e0180140. doi: 10.1371/journal.pone.0180140 (PMC5519043; doi:10.1371/journal.pone.0180140)
Supplement: S2 Table — (DOCX) [file pone.0180140.s002.docx]

**Table S2. Effect of categories of HIV RNA load at second-line ART initiation on time in years to second-line ART virological failure at 10^th^, 20^th^, 30^th^, 40^th^ and median survival time (Sweden 1999-2015, n=869).**

| **VL at second-line ART initiation** | **10th survival percentile** | **20th survival percentile** | **30th survival percentile** | **40th survival percentile** | **Median survival percentile** |
| --- | --- | --- | --- | --- | --- |
| **0-200** | 2.43a | 3.3 | 4 | 4.24 | 4.68 |
| **200-500** | -0.24  (-1.39; 0.9) b | -0.65  (-1.53; 0.23) | -0.77  (-1.45; -0.09) | -0.78  (-1.53; -0.02) | -0.82  (-1.51; -0.13) |
| **501-1000** | -0.54  (-1.95; 0.86) | -1.1  (-1.97; -0.23) | -1.43  (-2.19; -0.68) | -1.48  (-2.28; -0.68) | -1.52  (-2.27; -0.78) |
| **1001-10000** | -0.58  (-1.61; 0.45) | -0.86  (-1.59; -0.14) | -1.11  (-1.86; -0.35) | -1.16  (-2.2; -0.12) | -1.16  (-1.98; -0.33) |
| **10001-100000** | -0.61  (-3.48; 2.26) | -0.82  (-2.32; 0.68) | -1.1  (-2.01; -0.18) | -1.44  (-2.43; -0.45) | -1.56  (-2.81; -0.32) |
| **>100000** | -0.41  (-2.32; 1.5) | -0.84  (-6; 4.32) | -1.01  (-2.5; 0.47) | -1.02  (-2.04; 0) | -0.96  (-1.85; -0.08) |

Intercept coefficient with time in second-line ART and time in follow up centered in their mean. All the remaining covariates equal to reference group. Adjusted by: sex, type of therapeutic switch, type of regimen first and second-line ART, route of transmission, country of birth, age at first-line ART initiation, CD4 cell count at first and second-line ART initiation, HIV RNA load at first-line ART initiation, time in first-line ART, time in second-line ART and time in follow-up
