# Supplementary material for: Effect of therapy switch on time to second-line antiretroviral treatment failure in HIV-infected patients
Source: PLoS One. 2017 Jul 20;12(7):e0180140. doi: 10.1371/journal.pone.0180140 (PMC5519043; doi:10.1371/journal.pone.0180140)
Supplement: S4 Table — (DOCX) [file pone.0180140.s004.docx]

**Table S4. Estimates of the effect of viral load at second line ART initiation on CD4 cell count at 12 and 24 months (Sweden 1999-2015, N at 12 months=286, N at 24 month=202)**

|  | **Month** | **Median CD4 cell count quantile** |
| --- | --- | --- |
| **Therapeutic switch** |  |  |
| **0-200 (reference)** | **12** | 237 |
|  | **24** | 377 |
|  |  |  |
| **201-500** | **12** | 41 (-72;153) |
|  | **24** | 95 (-64;253) |
|  |  |  |
| **501-1,000** | **12** | 128 (-6;261) |
|  | **24** | 91 (-124;307) |
|  |  |  |
| **1,001-10,000** | **12** | -131 (-243;-18) |
|  | **24** | 42 (-161;245) |
|  |  |  |
| **10,001-100,000** | **12** | 1 (-167;170) |
|  | **24** | 81 (-281;443) |
|  |  |  |
| **100,000+** | **12** | -141 (-305;23) |
|  | **24** | 81 (-226;387) |

Intercept coefficient with time in second line ART and time in follow up centered in their mean. All the remaining covariates equal to reference group.Adjusted by: type of therapeutic switch, sex, indicator of virologic failure in second line ART, type of regime first and second line ART, route of transmission, country of birth. age at first line ART initiation, CD4 at first and second line ART initiation, VL RNA at first and second line ART initiation, time in first line ART, time in second line ART and time in follow up.
